# P27 Rezafungin treatment of candidaemia and invasive candidiasis: outcomes stratified by baseline renal function—analysis of the Phase 2 + Phase 3 trials

**DOI:** 10.1093/jacamr/dlad066.031

**Published:** 2023-06-14

**Authors:** Taylor Sandison, Jose A Vazquez, Patrick M Honore, Alex Soriano, Juan P Horcajada, Monica Slavin

**Affiliations:** Cidara Therapeutics Inc., San Diego, CA, USA; Augusta University, Augusta, GA, USA; Brugman University Hospital, Brussels, Belgium; Hospital Clínic, CIBERINFEC, University of Barcelona, Barcelona, Spain; Hospital Del Mar-IMIM, CIBERINFEC, Barcelona, Spain; Peter MacCallum Cancer Centre and Royal Melbourne Hospital, Melbourne, Australia

## Abstract

**Background:**

Rezafungin (RZF) once weekly (QWk) is a next-generation echinocandin in development for treatment of candidaemia and invasive candidiasis (IC) and prevention of invasive fungal disease caused by *Candida*, *Aspergillus*, and *Pneumocystis* spp. in BMT. RZF QWk was compared with caspofungin (CAS) QD in two double-blind, randomized, controlled trials of treatment of candidaemia and/or IC: STRIVE (Phase 2, NCT02734862) and ReSTORE (NCT03667690).

**Objectives:**

Trial data (Phase 2 + Phase 3) were analysed to evaluate outcomes stratified by renal function at baseline: CrCl ≥ 60 mL/min (normal/mild impairment [Norm/Mild]) and <60 mL/min (moderate/severe impairment [Mod/Sev]).

**Methods:**

Outcomes were evaluated for differences between CrCl categories and between treatment groups: RZF QWk 400 mg on Wk 1 then 200 mg versus CAS QD 70 mg on Day (D)1 then 50 mg, for ≥14 days (≤4 Wks) w/optional oral fluconazole stepdown for CAS.

**Results:**

D30 all-cause mortality (ACM)—Mod/Sev: RZF, 13% (7/54); CAS, 30.5% (18/59); Norm/Mild: RZF, 22.7% (17/75); CAS, 10.8% (9/83). Mycological eradication (ME) at D5—Mod/Sev: RZF, 75.9% (41/54); CAS, 61.0% (36/59); Norm/Mild: RZF, 74.7% (56/75); CAS, 66.3% (55/83). ME at D14—Mod/Sev: RZF, 75.9% (41/54); CAS, 57.6% (34/59); Norm/Mild: RZF, 69.3% (52/75); CAS, 74.7% (62/83). ≥1 treatment-emergent AE—Mod/Sev: RZF, 93.2% (55/59); CAS, 88.9% (56/63); Norm/Mild: RZF, 88.9% (72/81); CAS, 76.7% (69/90).

**Conclusions:**

RZF efficacy was comparable across CrCl categories, with higher ME and lower D30 ACM in the Mod/Sev group. Further analyses are needed to evaluate the observed differences between treatment groups.

